# The genome sequence of a cockchafer, *Melolontha melolontha* (Linnaeus, 1758)

**DOI:** 10.12688/wellcomeopenres.19434.1

**Published:** 2023-05-17

**Authors:** Mike Ashworth, Darwin Tree of Life Barcoding collective, Wellcome Sanger Institute Tree of Life programme, Wellcome Sanger Institute Scientific Operations: DNA Pipelines collective, Tree of Life Core Informatics collective, Darwin Tree of Life Consortium

**Affiliations:** 1 Independent researcher, Yeovil, England, UK

**Keywords:** Melolontha melolontha, a Cockchafer, genome sequence, chromosomal, Coleoptera

## Abstract

We present a genome assembly from an individual male *Melolontha melolontha* (a cockchafer; Arthropoda; Insecta; Coleoptera; Scarabaeidae). The genome sequence is 1,656.9 megabases in span. Most of the assembly is scaffolded into 10 chromosomal pseudomolecules, including the X sex chromosome. The mitochondrial genome has also been assembled and is 18.4 kilobases in length. Gene annotation of this assembly on Ensembl identified 17,392 protein coding genes.

## Species taxonomy

Eukaryota; Metazoa; Ecdysozoa; Arthropoda; Hexapoda; Insecta; Pterygota; Neoptera; Endopterygota; Coleoptera; Polyphaga; Scarabaeiformia; Scarabaeidae; Melolonthinae; *Melolontha*; *Melolontha melolontha* (Linnaeus, 1758) (NCBI:txid7061).

## Background

The beetle *Melolontha melolontha* (Linnaeus, 1758) (Coleoptera: Scarabaeidae) is one of several members of the genus commonly known in English as the Cockchafer or May Bug. The name ‘melolontha’ originates from the ancient Greek for ‘fig-sheep’ because of the tendency for the beetle to feed on wild figs. It is widely distributed across Europe from the west coast to Ukraine and Turkey in the east, and as far north as southern Scandinavia. It was formerly found in large numbers. Populations have been drastically reduced due to changes in land use and the widespread use of insecticides but have been recovering since the 1980s. In the United Kingdom, it remains locally common in England and Wales with a few scattered records in Scotland.

**Figure 1.**
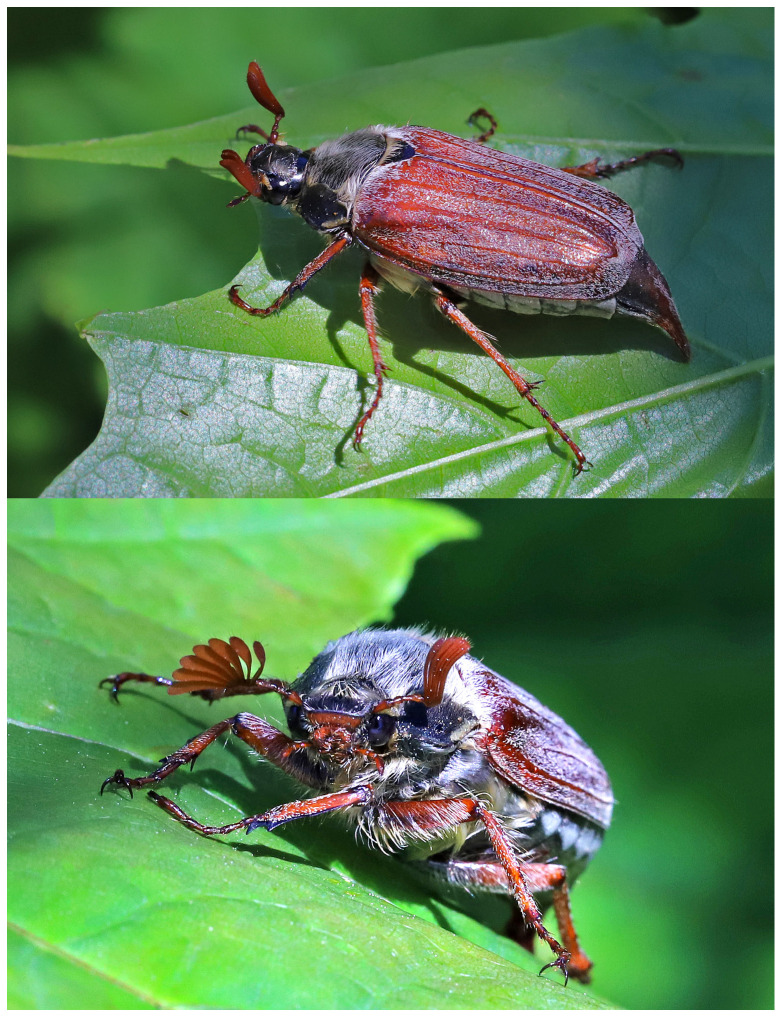
*Melolontha melolontha* (Linnaeus, 1758), European Cockchafer, to light trap, Yeovil, Somerset, United Kingdom, 30/31 May 2021. Photograph by Mike Ashworth.

Cockchafers are large and distinctive enough to make an impression in the public consciousness ( Figure 1), appearing in popular art, including paintings, opera, postcards, greeting cards and stamps, and as novelty chocolates ( Jones, 2018).

The larvae of *M. melolontha* feed on roots, taking about three years to develop to pupation. They can cause heavy damage to grasslands, fruit plantations and vineyards. In addition, the adults feed voraciously on the leaves of broadleaf trees, usually oak. In Central Europe the species has been regarded as an agricultural and horticultural pest. A range of control methods have been applied ( Malusá *et al.*, 2020), including the use of biological control agents, such as the entomopathogenic fungus *Beauveria brongniartii* ( Kessler *et al.*, 2004) and nematodes ( Erbaş *et al.*, 2014).

The specimen used for genome assembly was an adult male. Male cockchafers are known to be strongly attracted to light, and this one flew into a dwelling one warm spring evening on the 20 May 2020 in a rural village in Somerset, south-west England. The high-quality genome sequence for a male *M. melolontha* reported here has been generated as part of the Darwin Tree of Life project. It will aid in understanding the biology, physiology and ecology of the species.

## Genome sequence report

The genome was sequenced from one male *Melolontha melolontha* specimen collected from Yeovil, Somerset, UK (latitude 50.97, longitude –2.68). A total of 33-fold coverage in Pacific Biosciences single-molecule HiFi long reads was generated. Primary assembly contigs were scaffolded with chromosome conformation Hi-C data. Manual assembly curation corrected eight missing joins or mis-joins and removed four haplotypic duplications, reducing the assembly length by 0.15% and the scaffold number by 8.33%, and increasing the scaffold N50 by 1.14%.

**Figure 2.**
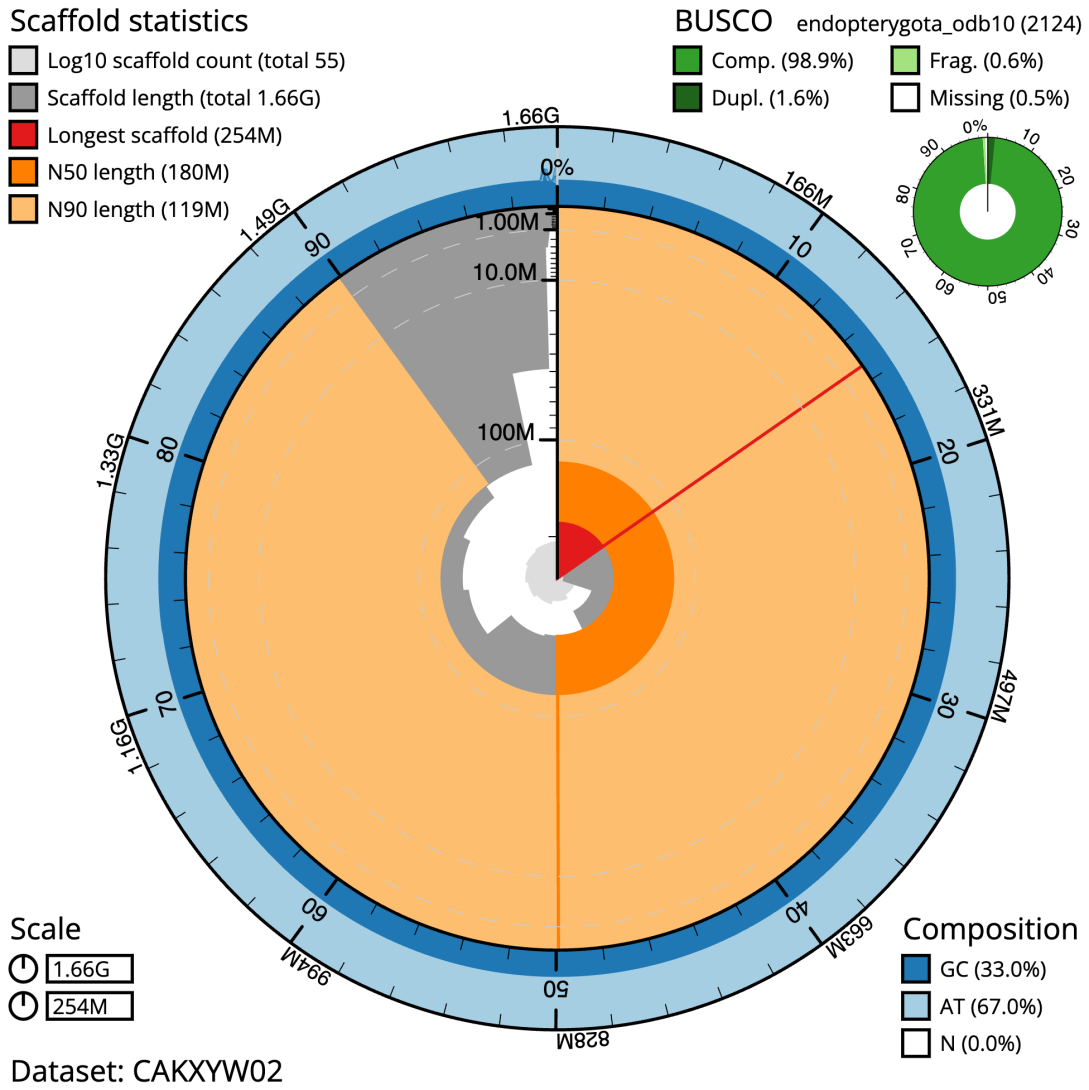
Genome assembly of *Melolontha melolontha*, icMelMelo1.2: metrics. The BlobToolKit Snailplot shows N50 metrics and BUSCO gene completeness. The main plot is divided into 1,000 size-ordered bins around the circumference with each bin representing 0.1% of the 1,656,884,372 bp assembly. The distribution of scaffold lengths is shown in dark grey with the plot radius scaled to the longest scaffold present in the assembly (253,604,678 bp, shown in red). Orange and pale-orange arcs show the N50 and N90 scaffold lengths (180,468,607 and 119,236,436 bp), respectively. The pale grey spiral shows the cumulative scaffold count on a log scale with white scale lines showing successive orders of magnitude. The blue and pale-blue area around the outside of the plot shows the distribution of GC, AT and N percentages in the same bins as the inner plot. A summary of complete, fragmented, duplicated and missing BUSCO genes in the endopterygota_odb10 set is shown in the top right. An interactive version of this figure is available at https://blobtoolkit.genomehubs.org/view/icMelMelo1.2/dataset/CAKXYW02/snail.

The final assembly has a total length of 1,656,9 Mb in 55 sequence scaffolds with a scaffold N50 of 180.5 Mb ( Table 1). Most (99.46%) of the assembly sequence was assigned to 10 chromosomal-level scaffolds, representing 9 autosomes and the X sex chromosome. Chromosome-scale scaffolds confirmed by the Hi-C data are named in order of size ( Figure 2– Figure 5; Table 2). While not fully phased, the assembly deposited is of one haplotype. Contigs corresponding to the second haplotype have also been deposited. The mitochondrial genome was also assembled and can be found as a contig within the multifasta file of the genome submission.

**Table 1.** Genome data for *Melolontha melolontha*, icMelMelo1.2.

Project accession data		
Assembly identifier	icMelMelo1.2	
Species	*Melolontha melolontha*	
Specimen	icMelMelo1	
NCBI taxonomy ID	7061	
BioProject	PRJEB50973	
BioSample ID	SAMEA7524378	
Isolate information	icMelMelo1, male; thorax (DNA sequencing); abdomen (RNA sequencing), head and thorax (Hi-C scaffolding)	
Assembly metrics *		*Benchmark*
Consensus quality (QV)	60.8	*≥ 50*
*k*-mer completeness	100%	*≥ 95%*
BUSCO **	C:98.9%[S:97.4%,D:1.6%], F:0.6%,M:0.5%,n:2,124	*C ≥ 95%*
Percentage of assembly mapped to chromosomes	99.46%	*≥ 95%*
Sex chromosomes	X chromosome	*localised homologous pairs*
Organelles	Mitochondrial genome assembled.	*complete single alleles*
Raw data accessions		
PacificBiosciences SEQUEL II	ERR8705878–ERR8705880	
Hi-C Illumina	ERR8702810, ERR8702811, ERR8702812	
PolyA RNA-Seq Illumina	ERR10378008	
Genome assembly		
Assembly accession	GCA_935421215.2	
*Accession of alternate haplotype*	GCA_935421255.1	
Span (Mb)	1,656.9	
Number of contigs	563	
Contig N50 length (Mb)	5.9	
Number of scaffolds	55	
Scaffold N50 length (Mb)	180.5	
Longest scaffold (Mb)	253.6	
Genome annotation		
Number of protein-coding genes	17,392	
Number of non-coding genes	4,888	
Number of gene transcripts	33,543	

* Assembly metric benchmarks are adapted from column VGP-2020 of “Table 1: Proposed standards and metrics for defining genome assembly quality” from ( Rhie *et al.*, 2021). ** BUSCO scores based on the endopterygota_odb10 BUSCO set using v5.3.2. C = complete [S = single copy, D = duplicated], F = fragmented, M = missing, n = number of orthologues in comparison. A full set of BUSCO scores is available at https://blobtoolkit.genomehubs.org/view/icMelMelo1.2/dataset/CAKXYW02/busco.

**Figure 3.**
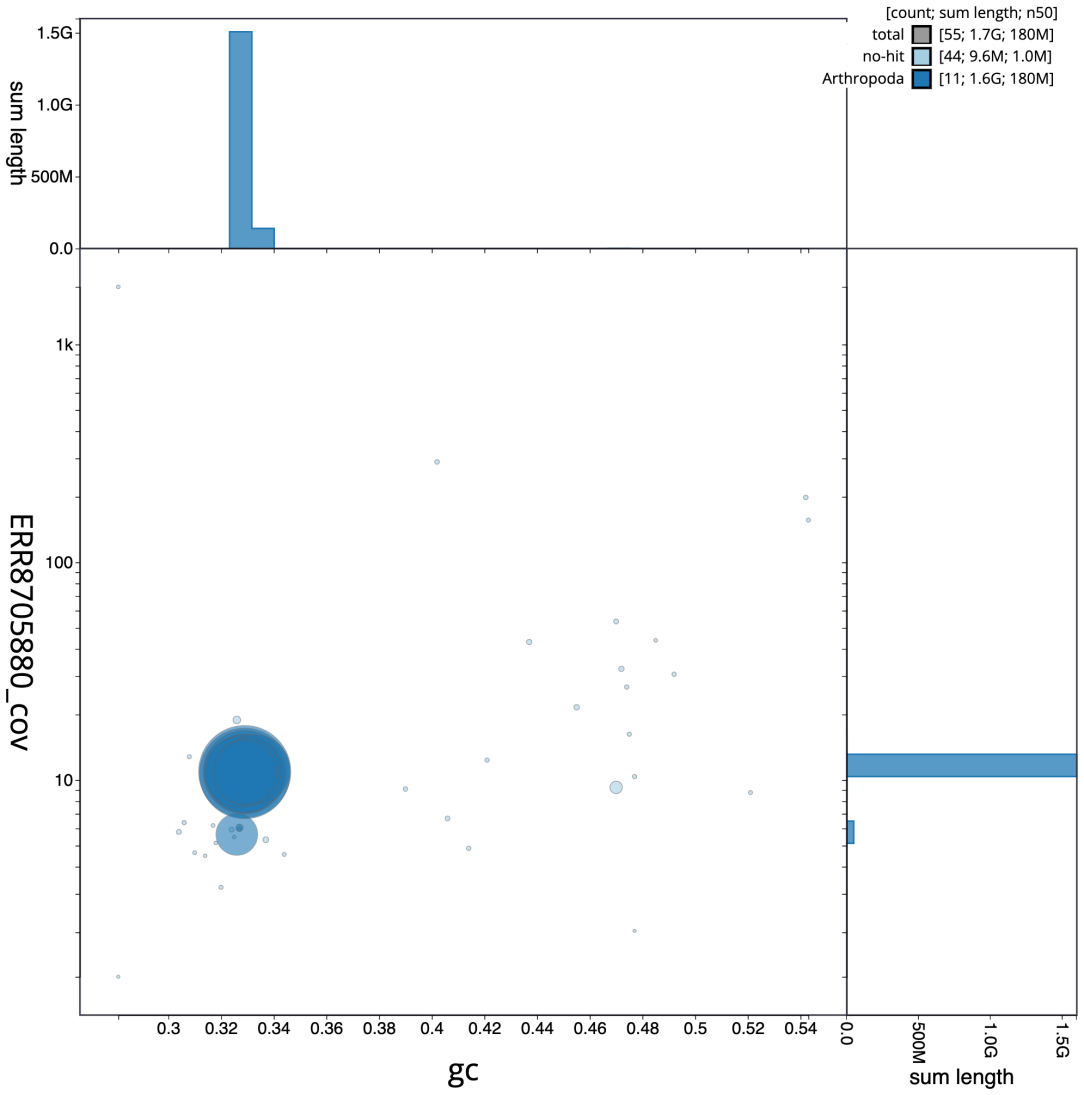
Genome assembly of *Melolontha melolontha*, icMelMelo1.2: BlobToolKit GC-coverage plot. Scaffolds are coloured by phylum. Circles are sized in proportion to scaffold length. Histograms show the distribution of scaffold length sum along each axis. An interactive version of this figure is available at https://blobtoolkit.genomehubs.org/view/icMelMelo1.2/dataset/CAKXYW02/blob.

**Figure 4.**
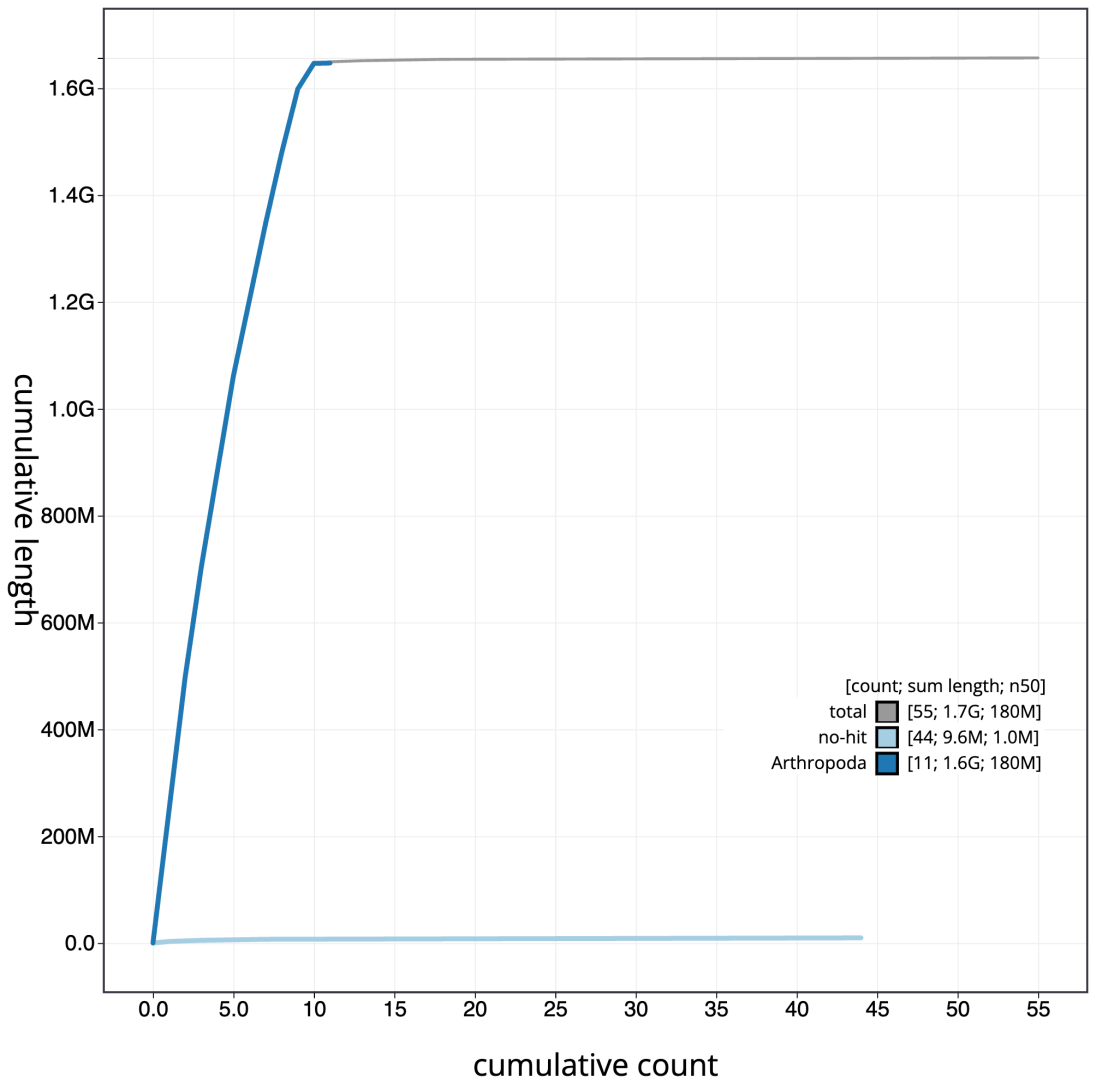
Genome assembly of *Melolontha melolontha*, icMelMelo1.2: BlobToolKit cumulative sequence plot. The grey line shows cumulative length for all scaffolds. Coloured lines show cumulative lengths of scaffolds assigned to each phylum using the buscogenes taxrule. An interactive version of this figure is available at https://blobtoolkit.genomehubs.org/view/icMelMelo1.2/dataset/CAKXYW02/cumulative.

**Figure 5.**
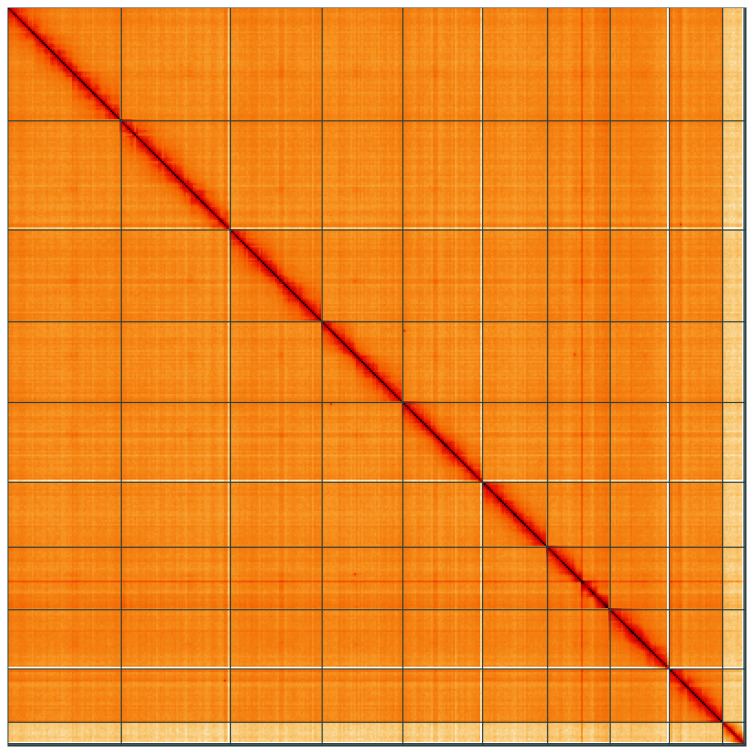
Genome assembly of *Melolontha melolontha*, icMelMelo1.2: Hi-C contact map of the icMelMelo1.2 assembly, visualised using HiGlass. Chromosomes are shown in order of size from left to right and top to bottom. An interactive version of this figure may be viewed at https://genome-note-higlass.tol.sanger.ac.uk/l/?d=WECRWt5cQgWcBCspo4Qc0g.

**Table 2.** Chromosomal pseudomolecules in the genome assembly of *Melolontha melolontha*, icMelMelo1.

INSDC accession	Chromosome	Size (Mb)	GC%
OW285235.1	1	253.61	32.9
OW285236.1	2	244.18	32.9
OW285237.1	3	205.25	32.9
OW285238.1	4	180.47	32.9
OW285239.1	5	178.43	32.9
OW285240.1	6	145.42	32.7
OW285241.1	7	139.93	33.3
OW285242.1	8	132.34	33
OW285243.1	9	119.24	32.9
OW285244.1	X	47.79	32.6
OW285245.2	MT	0.02	28.3
-	unplaced	10.22	38.7

The estimated Quality Value (QV) of the final assembly is 60.8 with *k*-mer completeness of 100%, and the assembly has a BUSCO v5.3.2 completeness of 98.9% (single = 97.4%, duplicated = 1.6%), using the endopterygota_odb10 reference set ( *n* = 2,124).

Metadata for specimens, spectral estimates, sequencing runs, contaminants and pre-curation assembly statistics can be found at https://links.tol.sanger.ac.uk/species/7061.

## Genome annotation report

The *M. melolontha* genome assembly (GCA_935421215.1) was annotated using the Ensembl rapid annotation pipeline ( Table 1; https://rapid.ensembl.org/Melolontha_melolontha_GCA_935421215.1/Info/Index). The resulting annotation includes 33,543 transcribed mRNAs from 17,392 protein-coding and 4,888 non-coding genes.

## Methods

### Sample acquisition and nucleic acid extraction

A male *Melolontha melolontha* specimen (icMelMelo1) was collected from Yeovil, Somerset, UK (latitude 50.97, longitude –2.68) on 20 May 2020. The specimen came to light from a rural garden and was collected by Mike Ashworth (independent researcher). The specimen was identified by the collector and preserved on dry ice.

The sample was prepared and DNA was extracted at the Tree of Life laboratory, Wellcome Sanger Institute (WSI). The icMelMelo1 sample was weighed and dissected on dry ice with tissue set aside for Hi-C sequencing. Thorax tissue was disrupted using a Nippi Powermasher fitted with a BioMasher pestle. High molecular weight (HMW) DNA was extracted using the Qiagen MagAttract HMW DNA extraction kit. HMW DNA was sheared into an average fragment size of 12–20 kb in a Megaruptor 3 system with speed setting 30. Sheared DNA was purified by solid-phase reversible immobilisation using AMPure PB beads with a 1.8X ratio of beads to sample to remove the shorter fragments and concentrate the DNA sample. The concentration of the sheared and purified DNA was assessed using a Nanodrop spectrophotometer and Qubit Fluorometer and Qubit dsDNA High Sensitivity Assay kit. Fragment size distribution was evaluated by running the sample on the FemtoPulse system.

RNA was extracted from abdomen tissue of icMelMelo1 in the Tree of Life Laboratory at the WSI using TRIzol, according to the manufacturer’s instructions. RNA was then eluted in 50 μl RNAse-free water and its concentration assessed using a Nanodrop spectrophotometer and Qubit Fluorometer using the Qubit RNA Broad-Range (BR) Assay kit. Analysis of the integrity of the RNA was done using Agilent RNA 6000 Pico Kit and Eukaryotic Total RNA assay.

### Sequencing

Pacific Biosciences HiFi circular consensus DNA sequencing libraries were constructed according to the manufacturers’ instructions. Poly(A) RNA-Seq libraries were constructed using the NEB Ultra II RNA Library Prep kit. DNA and RNA sequencing were performed by the Scientific Operations core at the WSI on Pacific Biosciences SEQUEL II (HiFi) and Illumina NovaSeq 6000 (RNA-Seq) instruments. Hi-C data were also generated from head and thorax tissue of icMelMelo1 using the Arima2 kit and sequenced on the HiSeq X Ten instrument.

### Genome assembly, curation and evaluation

Assembly was carried out with Hifiasm ( Cheng *et al.*, 2021) and haplotypic duplication was identified and removed with purge_dups ( Guan *et al.*, 2020). The assembly was then scaffolded with Hi-C data ( Rao *et al.*, 2014) using YaHS ( Zhou *et al.,* 2023). The assembly was checked for contamination as described previously ( Howe *et al.*, 2021). Manual curation was performed using HiGlass ( Kerpedjiev *et al.*, 2018) and Pretext ( Harry, 2022). The mitochondrial genome was assembled using MitoHiFi ( Uliano-Silva *et al.*, 2022), which runs MitoFinder ( Allio *et al.*, 2020) or MITOS ( Bernt *et al.*, 2013) and uses these annotations to select the final mitochondrial contig and to ensure the general quality of the sequence.

A Hi-C map for the final assembly was produced using bwa-mem2 ( Vasimuddin *et al.*, 2019) in the Cooler file format ( Abdennur & Mirny, 2020). To assess the assembly metrics, the *k*-mer completeness and QV consensus quality values were calculated in Merqury ( Rhie *et al.*, 2020). This work was done using Nextflow ( Di Tommaso *et al.*, 2017) DSL2 pipelines “sanger-tol/readmapping” ( Surana *et al.*, 2023a) and “sanger-tol/genomenote” ( Surana *et al.*, 2023b). The genome was analysed within the BlobToolKit environment ( Challis *et al.*, 2020) and BUSCO scores ( Manni *et al.*, 2021; Simão *et al.*, 2015) were calculated.

Table 3 contains a list of relevant software tool versions and sources.

**Table 3.** Software tools: versions and sources.

Software tool	Version	Source
BlobToolKit	4.0.7	https://github.com/blobtoolkit/blobtoolkit
BUSCO	5.3.2	https://gitlab.com/ezlab/busco
Hifiasm	0.16.1-r375	https://github.com/chhylp123/hifiasm
HiGlass	1.11.6	https://github.com/higlass/higlass
Merqury	MerquryFK	https://github.com/thegenemyers/MERQURY.FK
MitoHiFi	2	https://github.com/marcelauliano/MitoHiFi
PretextView	0.2	https://github.com/wtsi-hpag/PretextView
purge_dups	1.2.3	https://github.com/dfguan/purge_dups
sanger-tol/genomenote	v1.0	https://github.com/sanger-tol/genomenote
sanger-tol/readmapping	1.1.0	https://github.com/sanger-tol/readmapping/tree/1.1.0
YaHS	yahs-1.1.91eebc2	https://github.com/c-zhou/yahs

### Genome annotation

The Ensembl gene annotation system ( Aken *et al.*, 2016) was used to generate annotation for the *Melolontha melolontha* assembly (GCA_935421215.1). Annotation was created primarily through alignment of transcriptomic data to the genome, with gap filling via protein-to-genome alignments of a select set of proteins from UniProt ( UniProt Consortium, 2019).

### Ethics and compliance issues

The materials that have contributed to this genome note have been supplied by a Darwin Tree of Life Partner. The submission of materials by a Darwin Tree of Life Partner is subject to the Darwin Tree of Life Project Sampling Code of Practice. By agreeing with and signing up to the Sampling Code of Practice, the Darwin Tree of Life Partner agrees they will meet the legal and ethical requirements and standards set out within this document in respect of all samples acquired for, and supplied to, the Darwin Tree of Life Project. All efforts are undertaken to minimise the suffering of animals used for sequencing. Each transfer of samples is further undertaken according to a Research Collaboration Agreement or Material Transfer Agreement entered into by the Darwin Tree of Life Partner, Genome Research Limited (operating as the Wellcome Sanger Institute), and in some circumstances other Darwin Tree of Life collaborators.

## Data Availability

European Nucleotide Archive: *Melolontha melolontha* (cockchafer). Accession number PRJEB50973; https://identifiers.org/ena.embl/PRJEB50973 ( Wellcome Sanger Institute, 2022). The genome sequence is released openly for reuse. The *Melolontha melolontha* genome sequencing initiative is part of the Darwin Tree of Life (DToL) project. All raw sequence data and the assembly have been deposited in INSDC databases. Raw data and assembly accession identifiers are reported in Table 1.

## Author information

Members of the Darwin Tree of Life Barcoding collective are listed here: https://doi.org/10.5281/zenodo.4893703.

Members of the Wellcome Sanger Institute Tree of Life programme are listed here: https://doi.org/10.5281/zenodo.4783585.

Members of Wellcome Sanger Institute Scientific Operations: DNA Pipelines collective are listed here: https://doi.org/10.5281/zenodo.4790455.

Members of the Tree of Life Core Informatics collective are listed here: https://doi.org/10.5281/zenodo.5013541.

Members of the Darwin Tree of Life Consortium are listed here: https://doi.org/10.5281/zenodo.4783558.

## References

[ref-1] Abdennur N Mirny LA : Cooler: Scalable storage for Hi-C data and other genomically labeled arrays. *Bioinformatics.* 2020;36(1):311–316. 10.1093/bioinformatics/btz540 31290943 PMC8205516

[ref-2] Aken BL Ayling S Barrell D : The Ensembl gene annotation system. *Database (Oxford).* 2016;2016:baw093. 10.1093/database/baw093 27337980 PMC4919035

[ref-3] Allio R Schomaker-Bastos A Romiguier J : MitoFinder: Efficient automated large‐scale extraction of mitogenomic data in target enrichment phylogenomics. *Mol Ecol Resour.* 2020;20(4):892–905. 10.1111/1755-0998.13160 32243090 PMC7497042

[ref-4] Bernt M Donath A Jühling F : MITOS: Improved *de novo* metazoan mitochondrial genome annotation. *Mol Phylogenet Evol.* 2013;69(2):313–9. 10.1016/j.ympev.2012.08.023 22982435

[ref-5] Challis R Richards E Rajan J : BlobToolKit - interactive quality assessment of genome assemblies. *G3 (Bethesda).* 2020;10(4):1361–1374. 10.1534/g3.119.400908 32071071 PMC7144090

[ref-6] Cheng H Concepcion GT Feng X : Haplotype-resolved *de novo* assembly using phased assembly graphs with hifiasm. *Nat Methods.* 2021;18(2):170–175. 10.1038/s41592-020-01056-5 33526886 PMC7961889

[ref-22] Di Tommaso P Chatzou M Floden EW : Nextflow enables reproducible computational workflows. *Nat Biotechnol.* 2017;35(4):316–319. 10.1038/nbt.3820 28398311

[ref-7] Erbaş Z Gokce C Hazir S : Isolation and identification of entomopathogenic nematodes (Nematoda: Rhabditida) from the Eastern Black Sea region and their biocontrol potential against Melolontha melolontha (Coleoptera: Scarabaeidae) larvae. *Turk J Agric For.* 2014;38(2):187–197. 10.3906/tar-1301-42

[ref-8] Guan D McCarthy SA Wood J : Identifying and removing haplotypic duplication in primary genome assemblies. *Bioinformatics.* 2020;36(9):2896–2898. 10.1093/bioinformatics/btaa025 31971576 PMC7203741

[ref-9] Harry E : PretextView (Paired REad TEXTure Viewer): A desktop application for viewing pretext contact maps. 2022; (Accessed: 19 October 2022). Reference Source

[ref-10] Howe K Chow W Collins J : Significantly improving the quality of genome assemblies through curation. *GigaScience.* Oxford University Press,2021;10(1):giaa153. 10.1093/gigascience/giaa153 33420778 PMC7794651

[ref-11] Jones R : Beetles.Collins New Naturalist Library,2018.

[ref-12] Kerpedjiev P Abdennur N Lekschas F : HiGlass: Web-based visual exploration and analysis of genome interaction maps. *Genome Biol.* 2018;19(1):125. 10.1186/s13059-018-1486-1 30143029 PMC6109259

[ref-13] Kessler P Enkerl J Schweize C : Survival of *Beauveria brongniartii* in the soil after application as a biocontrol agent against the European cockchafer *Melolontha melolontha*. *BioControl.* 2004;49(5):563–581. 10.1023/B:BICO.0000036441.40227.ed

[ref-14] Malusá E Tartanus M Furmanczyk EM : Holistic approach to control *Melolontha* spp. in organic strawberry plantations. *Org Agr.* 2020;10(Suppl 1):13–22. 10.1007/s13165-020-00295-2

[ref-15] Manni M Berkeley MR Seppey M : BUSCO Update: Novel and Streamlined Workflows along with Broader and Deeper Phylogenetic Coverage for Scoring of Eukaryotic, Prokaryotic, and Viral Genomes. *Mol Biol Evol.* 2021;38(10):4647–4654. 10.1093/molbev/msab199 34320186 PMC8476166

[ref-16] Rao SSP Huntley MH Durand NC : A 3D map of the human genome at kilobase resolution reveals principles of chromatin looping. *Cell.* 2014;159(7):1665–80. 10.1016/j.cell.2014.11.021 25497547 PMC5635824

[ref-18] Rhie A McCarthy SA Fedrigo O : Towards complete and error-free genome assemblies of all vertebrate species. *Nature.* 2021;592(7856):737–746. 10.1038/s41586-021-03451-0 33911273 PMC8081667

[ref-17] Rhie A Walenz BP Koren S : Merqury: Reference-free quality, completeness, and phasing assessment for genome assemblies. *Genome Biology.* 2020;21(1):245. 10.1186/s13059-020-02134-9 32928274 PMC7488777

[ref-19] Simão FA Waterhouse RM Ioannidis P : BUSCO: assessing genome assembly and annotation completeness with single-copy orthologs. *Bioinformatics.* 2015;31(19):3210–2. 10.1093/bioinformatics/btv351 26059717

[ref-20] Surana P Muffato M Qi G : sanger-tol/readmapping: sanger-tol/readmapping v1.1.0 - Hebridean Black (1.1.0).Zenodo. 2023a; (Accessed: 17 April 2023). 10.5281/zenodo.7755665

[ref-21] Surana P Muffato M Sadasivan Baby C : sanger-tol/genomenote (v1.0.dev).Zenodo. 2023b; (Accessed: 17 April 2023). 10.5281/zenodo.6785935

[ref-23] Uliano-Silva M Ferreira JGRN Krasheninnikova K : MitoHiFi: a python pipeline for mitochondrial genome assembly from PacBio High Fidelity reads. *bioRxiv.* [Preprint],2022. 10.1101/2022.12.23.521667 PMC10354987 37464285

[ref-24] UniProt Consortium: UniProt: a worldwide hub of protein knowledge. *Nucleic Acids Res.* 2019;47(D1):D506–D515. 10.1093/nar/gky1049 30395287 PMC6323992

[ref-25] Vasimuddin Md Misra S Li H : Efficient Architecture-Aware Acceleration of BWA-MEM for Multicore Systems.In: *2019 IEEE International Parallel and Distributed Processing Symposium (IPDPS).*IEEE,2019;314–324. 10.1109/IPDPS.2019.00041

[ref-26] Wellcome Sanger Institute: The genome sequence of a cockchafer, *Melolontha melolontha* (Linnaeus, 1758). European Nucleotide Archive.[dataset], accession number PRJEB50973,2022.

[ref-27] Zhou C McCarthy SA Durbin R : YaHS: yet another Hi-C scaffolding tool. *Bioinformatics.* Edited by C. Alkan,2023;39(1):btac808. 10.1093/bioinformatics/btac808 36525368 PMC9848053

