# Supplementary material for: The genome sequence of a cockchafer, Melolontha melolontha (Linnaeus, 1758)
Source: Wellcome Open Res. 2023 May 17;8:222. [Version 1] doi: 10.12688/wellcomeopenres.19434.1 (PMC11015117; doi:10.12688/wellcomeopenres.19434.1)
Supplement: Peer review history

# Reviewer report: Reviewer response for version 1

**DOI:** 10.21956/wellcomeopenres.21530.r77530

**Published:** 2024-09-04

**Authors:** Lapo Ragionieri

**Affiliations:** 1 University of Cologne, Cologne, Germany

The data note titled "The genome sequence of a cockchafer, *Melolontha melolontha* (Linnaeus, 1758)" by Mike Ashworth describes the sequencing and genome assembly of *Melolontha melolontha*. The authors utilized long-read sequencing technology (PacBio) to produce an initial assembly, which was subsequently scaffolded using Hi-C, resulting in a genome assembly at the chromosome level, with nearly all scaffolds assigned to chromosomes. The genome sequence of the cockchafer, *Melolontha melolontha*, represents a significant contribution to the fields of entomology and genomics. The genome assembly has a total length of 1,656 Mb across 55 sequence scaffolds, with a scaffold N50 of 180.5 Mb. There are some discrepancies between the text and the tables that should be corrected. The genome annotation includes 33,543 transcribed mRNAs, comprising 17,392 protein-coding genes and 4,888 non-coding genes.

 The presentation of the results is well done, but the authors did not report any information about the masking of the genome.

**Below are some specific comments and suggestions:**

1. **Scale Bar**: A scale bar is missing in Figure 1.
2. **Keywords**: The keywords should be distinct from the title to be effective.
3. **Species Identification**: The authors should cite the literature used for species identification.
4. **Mitochondrial Genome**: I strongly recommend submitting the mitochondrial genome separately with an independent accession number. Moreover, while the authors described the methods, they did not provide information about the mitochondrial genome annotation (coding genes, rRNAs, and tRNAs). It is expected that the authors submit the annotations obtained by MITOS separately.
5. **Annotation**: The authors should identify and quantify the transposable elements in the genome.
6. **Command Lines**: It is common practice to include the command lines used with all software. Providing this information would be very useful for readers.

Are sufficient details of methods and materials provided to allow replication by others?

Yes

Is the rationale for creating the dataset(s) clearly described?

Yes

Are the datasets clearly presented in a useable and accessible format?

Partly

Are the protocols appropriate and is the work technically sound?

Yes

Reviewer Expertise:

Transcriptomic genomics proteomics

I confirm that I have read this submission and believe that I have an appropriate level of expertise to confirm that it is of an acceptable scientific standard, however I have significant reservations, as outlined above.

# Reviewer report: Reviewer response for version 1

**DOI:** 10.21956/wellcomeopenres.21530.r77527

**Published:** 2024-04-12

**Authors:** Yoshiaki Morino

**Affiliations:** 1 Institute of Life and Environmental Sciences, University of Tsukuba, Tsukuba, Japan

The authors present the genome sequence of a cockchafer, *Melolontha melolontha.* The constructed genome assembly was 1,656,9 Mb in 55 sequence scaffolds. Those sequences can be assigned to 10 chromosome-level scaffolds, including the X sex chromosome. Using Ensembl rapid annotation pipeline, 17,392 protein-coding genes were predicted. Assembly metrics meet benchmarks and are of sufficiently high quality. Therefore, this genomic data will facilitate the development of physiological and ecological studies of this or related species.

 I only have two minor comments as below.

 1. A description of what tissues and organs the "abdominal tissue" for RNA-seq actually contains would improve its future availability.

 2. In relation to the identification of sex chromosomes, I would recommend that information on the sex determination mechanisms or sex chromosomes of this or related species be described in the background.

Are sufficient details of methods and materials provided to allow replication by others?

Yes

Is the rationale for creating the dataset(s) clearly described?

Yes

Are the datasets clearly presented in a useable and accessible format?

Yes

Are the protocols appropriate and is the work technically sound?

Yes

Reviewer Expertise:

Marine invertebrate, Evo-Devo

I confirm that I have read this submission and believe that I have an appropriate level of expertise to confirm that it is of an acceptable scientific standard.

# Reviewer report: Reviewer response for version 1

**DOI:** 10.21956/wellcomeopenres.21530.r77529

**Published:** 2024-04-11

**Authors:** Beulah Garner

**Affiliations:** 1 Natural History Museum, London, England, UK

In this data note, the authors present the genome assembly of *Melolontha melolontha*, the European cockchafer. The assembly consist of 10 chromosome scaffolds which is concurrent with other studies of this kind within the Scarabaeoidea.

 The latest methods for genome sequencing, assembly, annotation and characterization used in this study are standardized across the Darwin Tree of Life Consortium, being clearly and methodically described here. Sequencing assembly is clearly supplemented with the appropriate illustrations. The standard for generating high quality genome assemblies is high, therefore this genome sequence is a valuable resource for studies into the systematics of this species as well as its relatives.

 As a curiosity, why was DNA and RNA extracted from different parts of the beetle (Johnson, et al., 2013 [Ref 1]).

 The title should be clear this is the European cockchafer and in fact a beetle (Coleoptera). Consistency in reporting the species name, author and year. Cite the original publication (Wägele, *et al*., 2011[Ref 2]). 

 I would like to see references for the statements on *M. melolontha* decline and for it's recovery since the 1980s to improve the overall rationale.

Are sufficient details of methods and materials provided to allow replication by others?

Yes

Is the rationale for creating the dataset(s) clearly described?

Yes

Are the datasets clearly presented in a useable and accessible format?

Yes

Are the protocols appropriate and is the work technically sound?

Yes

Reviewer Expertise:

Coleoptera taxonomy and systematics.

I confirm that I have read this submission and believe that I have an appropriate level of expertise to confirm that it is of an acceptable scientific standard.

## References

[1] : The importance of tissue specificity for RNA-seq: highlighting the errors of composite structure extractions. *BMC Genomics* .2013;14: 10.1186/1471-2164-14-586 586 10.1186/1471-2164-14-586 23985010 PMC3765781

[2] : The taxonomist - an endangered race. A practical proposal for its survival. *Front Zool* .2011;8(1) : 10.1186/1742-9994-8-25 25 10.1186/1742-9994-8-25 22029904 PMC3210083

# Reviewer report: Reviewer response for version 1

**DOI:** 10.21956/wellcomeopenres.21530.r77533

**Published:** 2024-03-27

**Authors:** Fahad Alqahtani

**Affiliations:** 1 King Abdulaziz City for Science and Technology, Riyadh, Saudi Arabia

The authors have achieved a highly complete genome assembly at the chromosome level and provided high-quality annotation for an adult male cockchafer, *Melolontha melolontha* (Linnaeus, 1758). The genome assembly utilized three sequencing technologies: Pacific Biosciences SEQUEL II, Hi-C Illumina, and PolyA RNA-Seq Illumina. The completeness of the genome assembly was assessed using BUSCO analysis, which indicated a genome size of 1,656.9 Mb, with 98.9% of common genes completely present. Figure 1 is very detailed, showcasing some features clearly.

 However, minor comments should be addressed:

- In the "Genome Sequence Report," the authors have incorrectly stated the total length of the final assembly as "1,656,9 Mb"; this should be corrected to 1,656.9 Mb.
- Also, in the "Genome Sequence Report" section, the authors state that the number of sequence scaffolds is 55, which differs from the 54 reported in NCBI. This difference may be due to the inclusion of the mitochondrial sequence; if so, the authors should clarify that the mitochondrial sequence is included. This adjustment should also be reflected in Table 1.
- In Table 1, the authors report the number of contigs as 563, while NCBI lists 562.
- "Pretext" in the Genome Assembly section should be corrected to "PretextView".

Are sufficient details of methods and materials provided to allow replication by others?

Yes

Is the rationale for creating the dataset(s) clearly described?

Yes

Are the datasets clearly presented in a useable and accessible format?

Yes

Are the protocols appropriate and is the work technically sound?

Yes

Reviewer Expertise:

Bioinformatics

I confirm that I have read this submission and believe that I have an appropriate level of expertise to confirm that it is of an acceptable scientific standard, however I have significant reservations, as outlined above.
